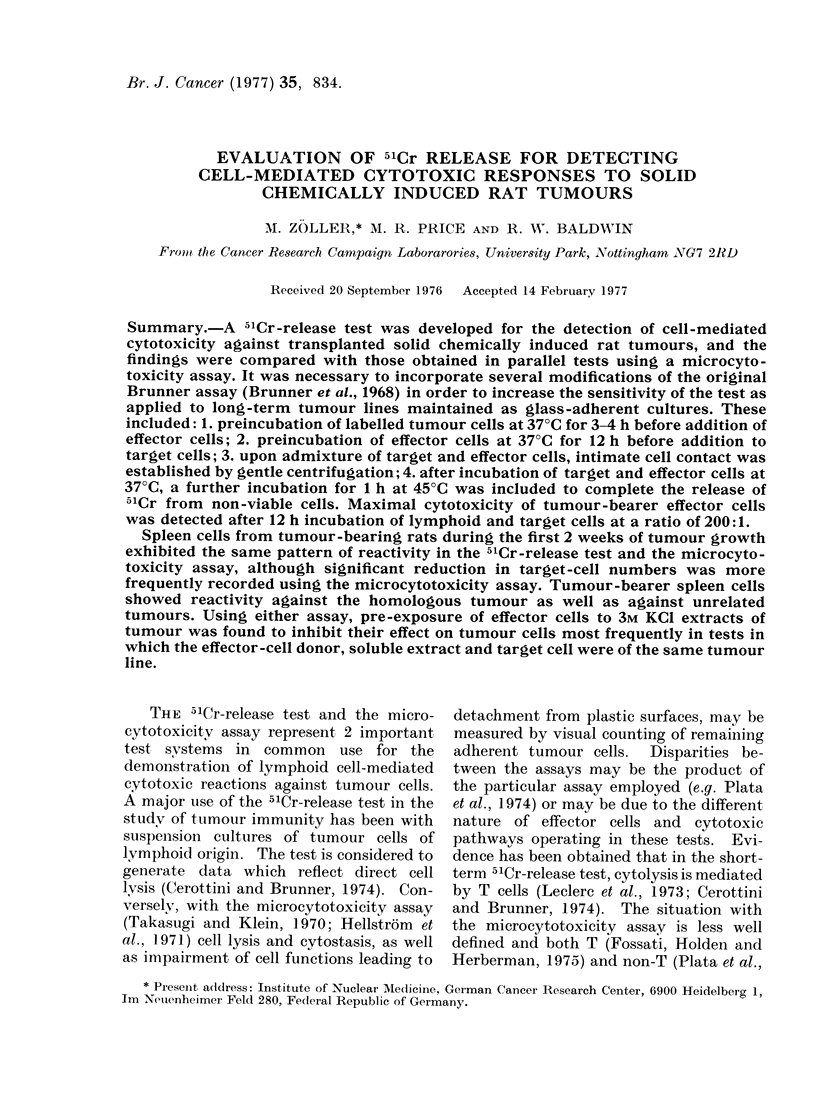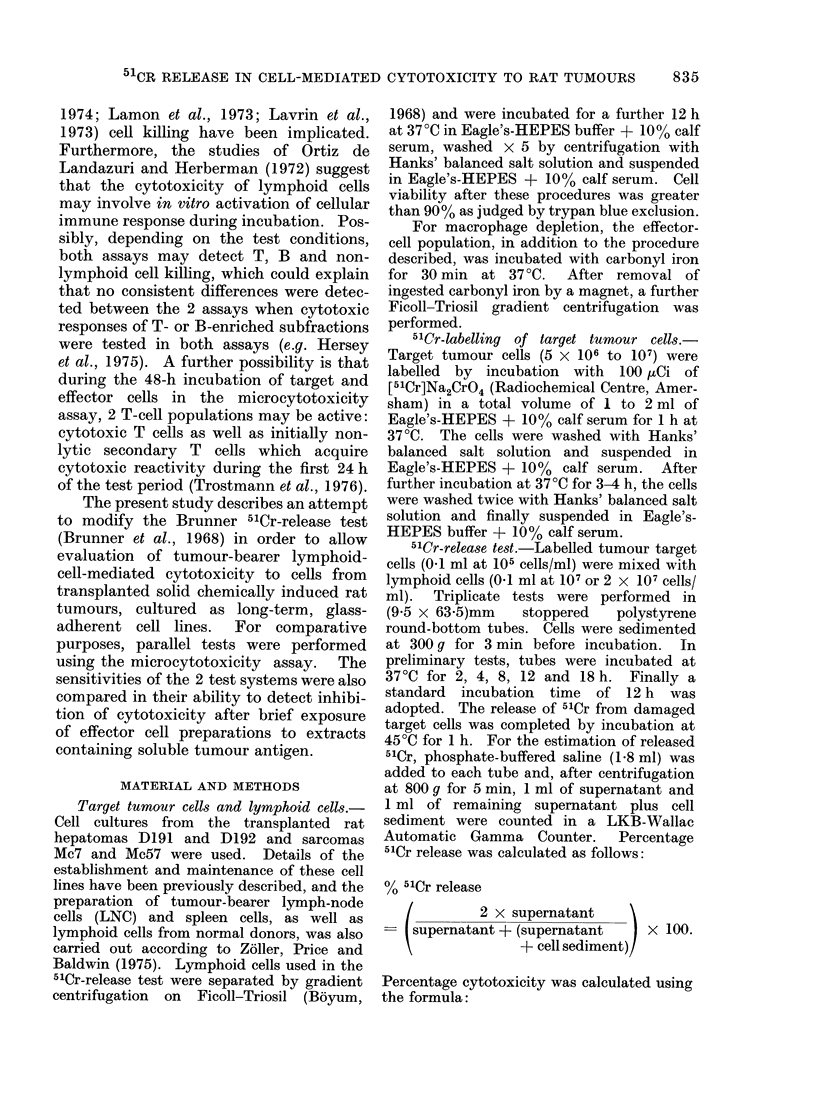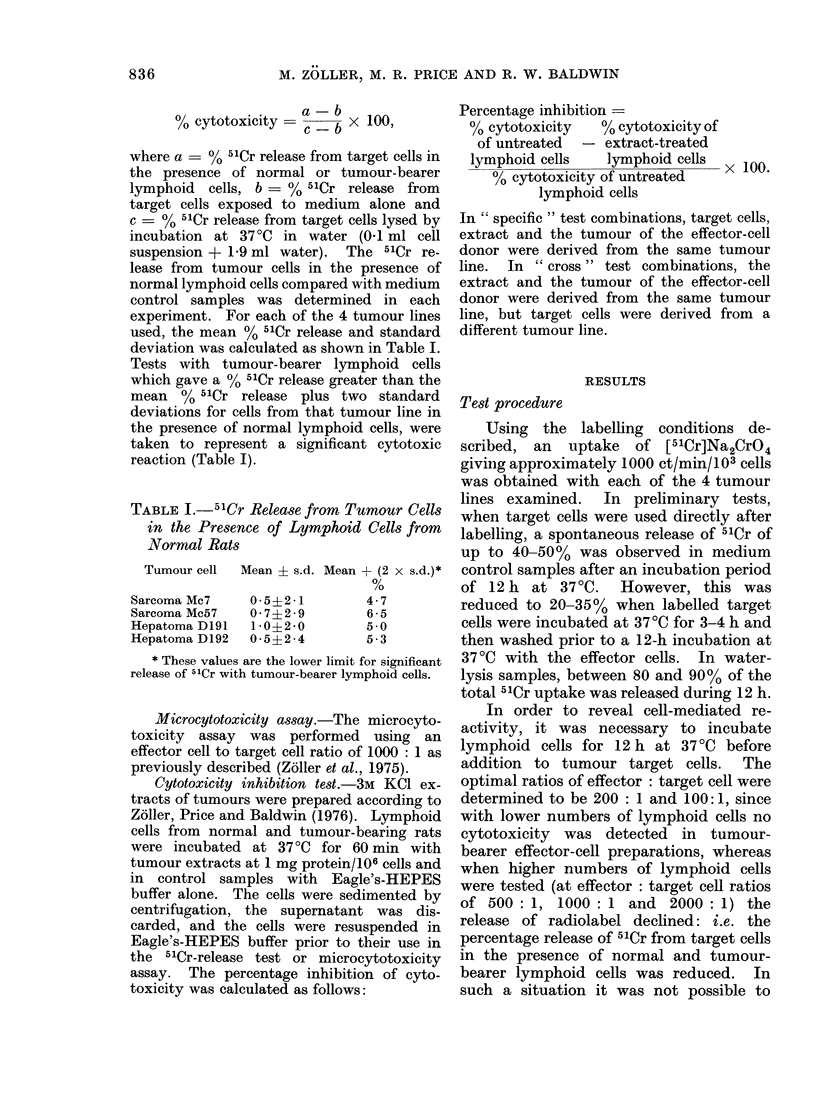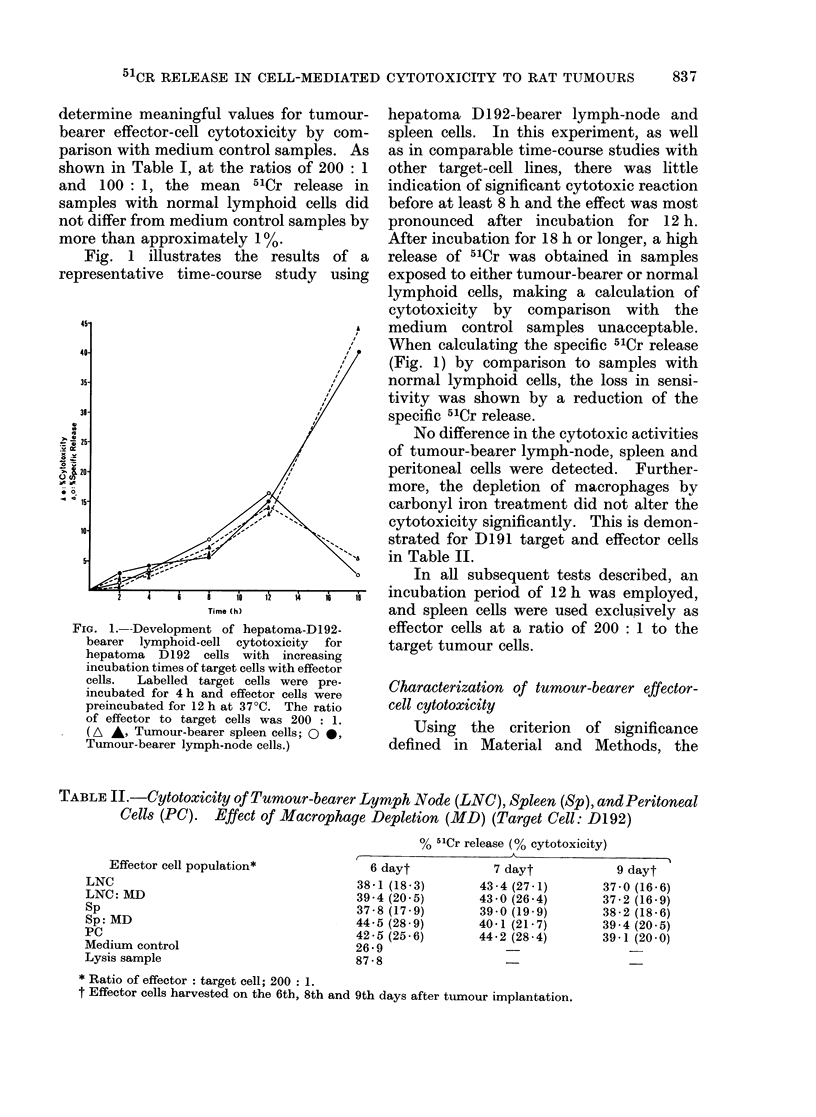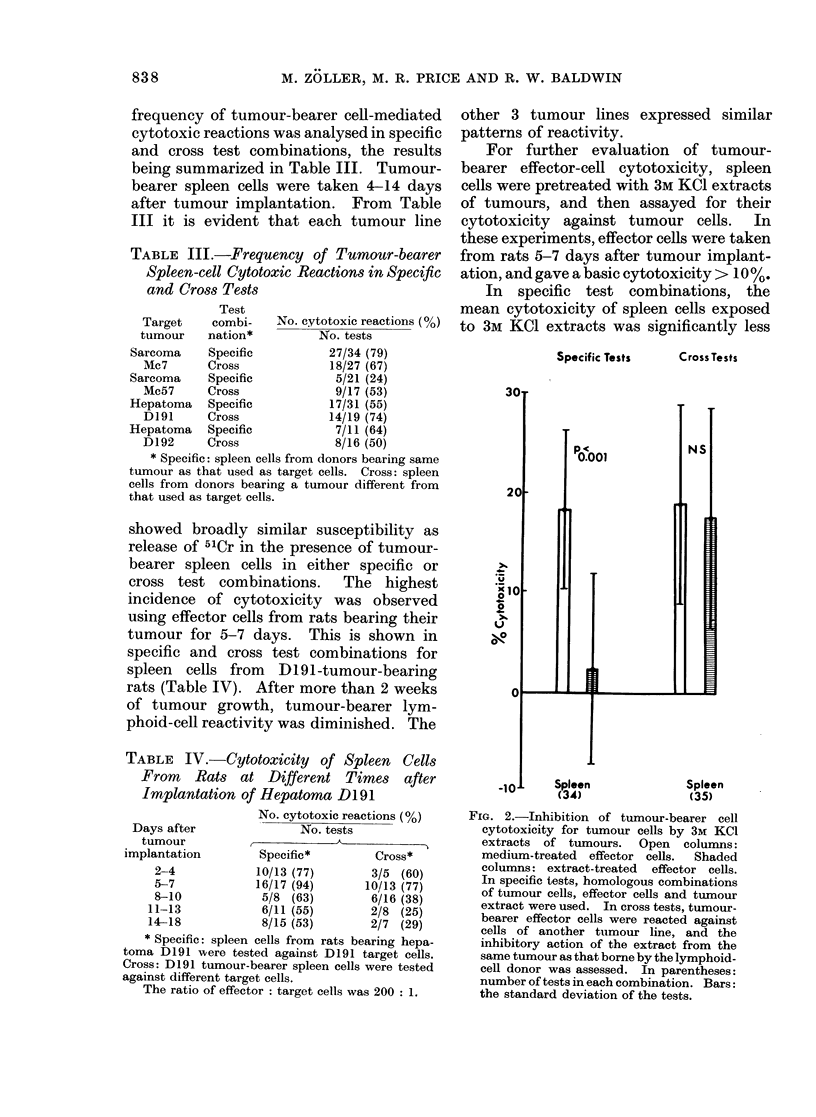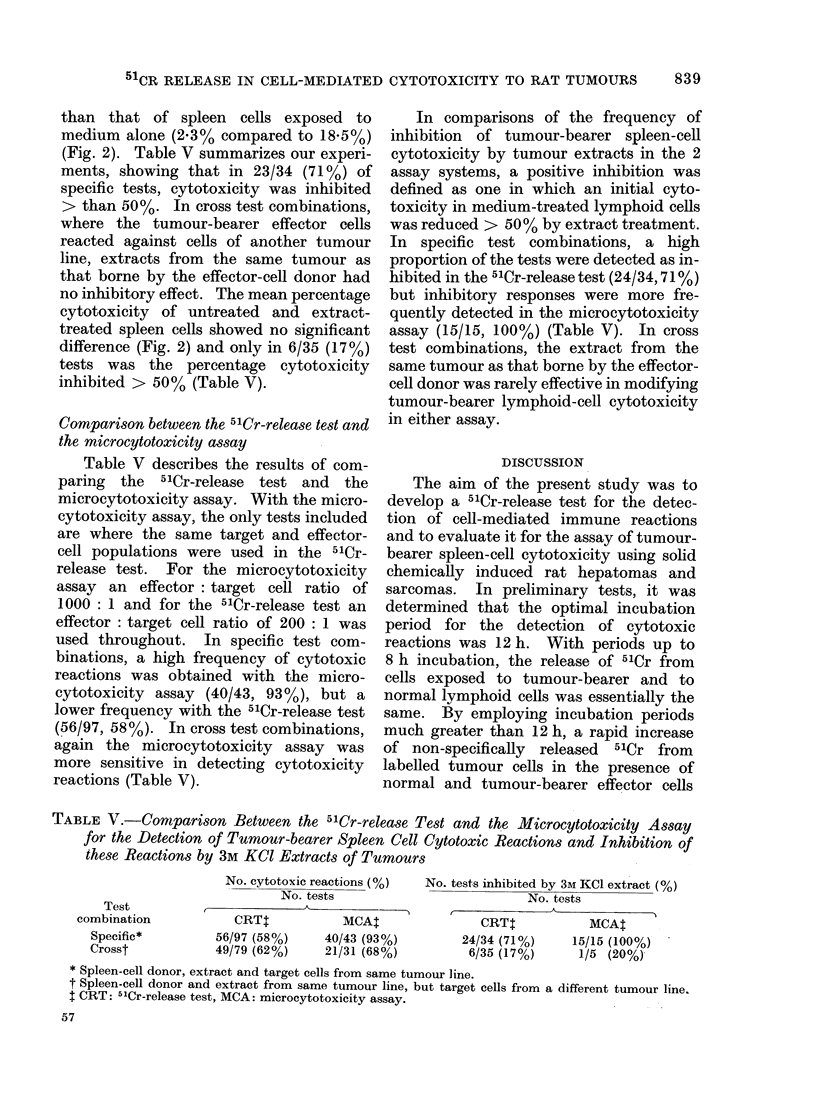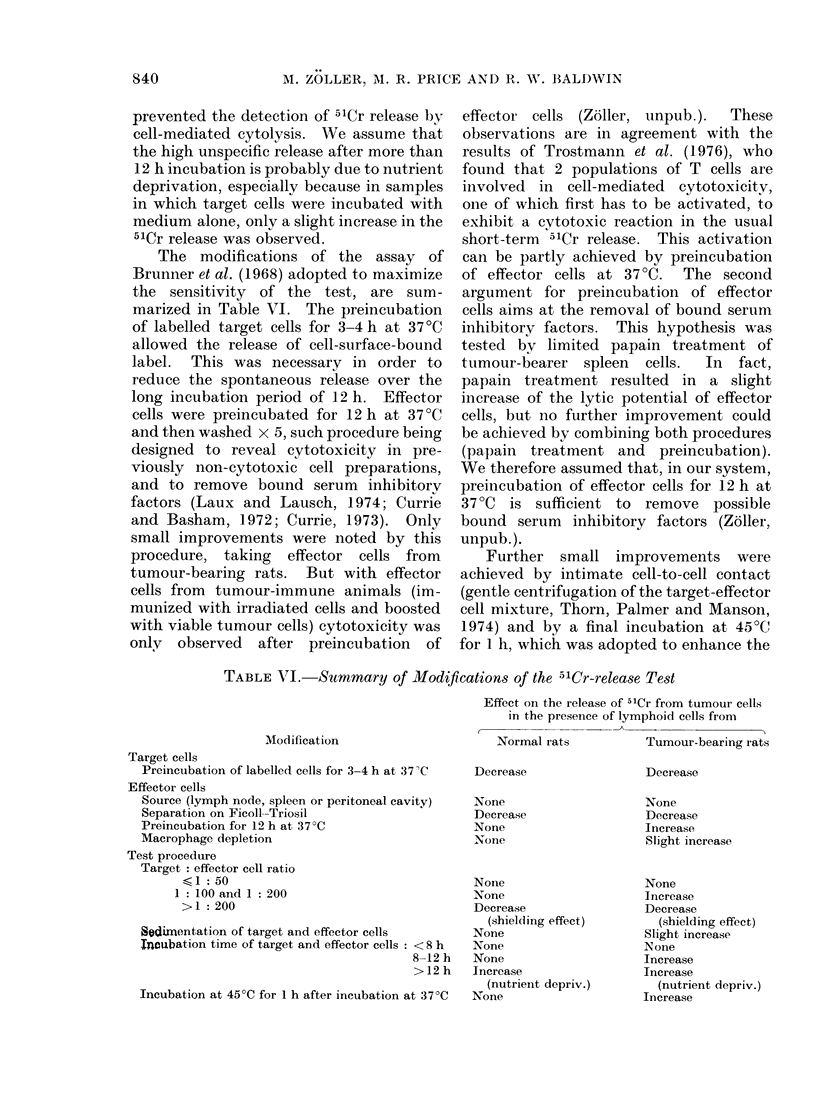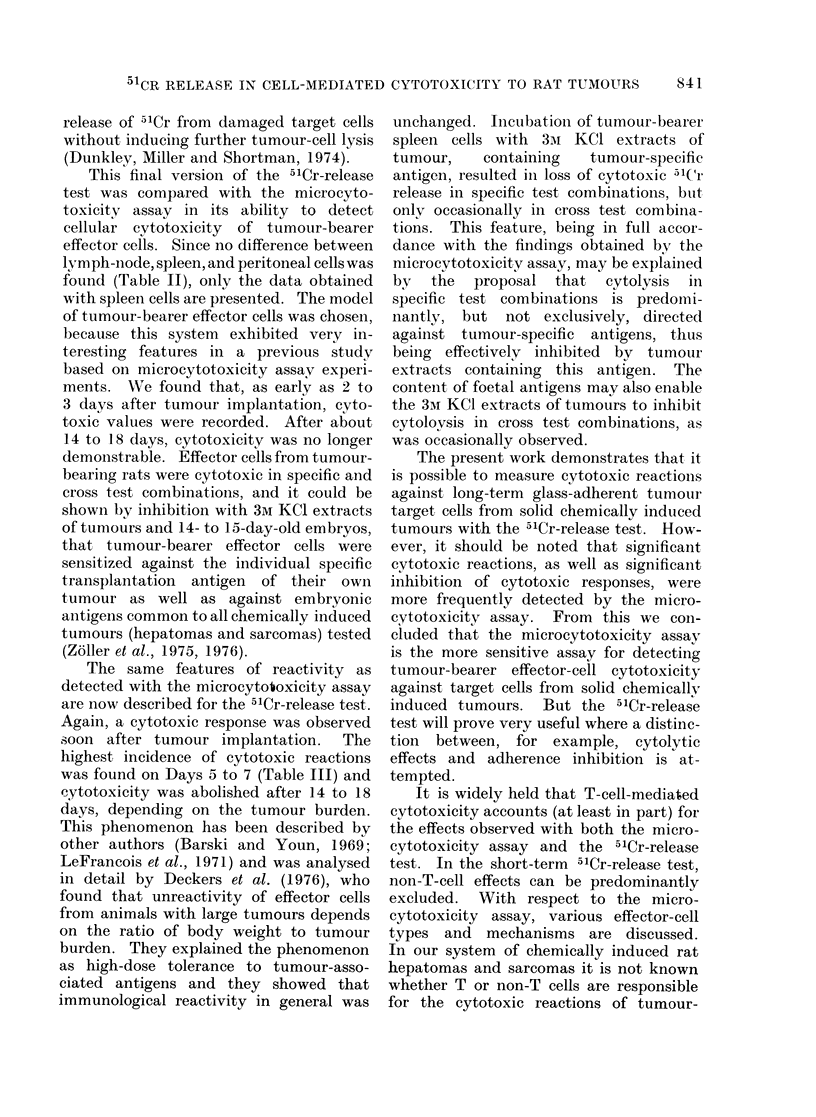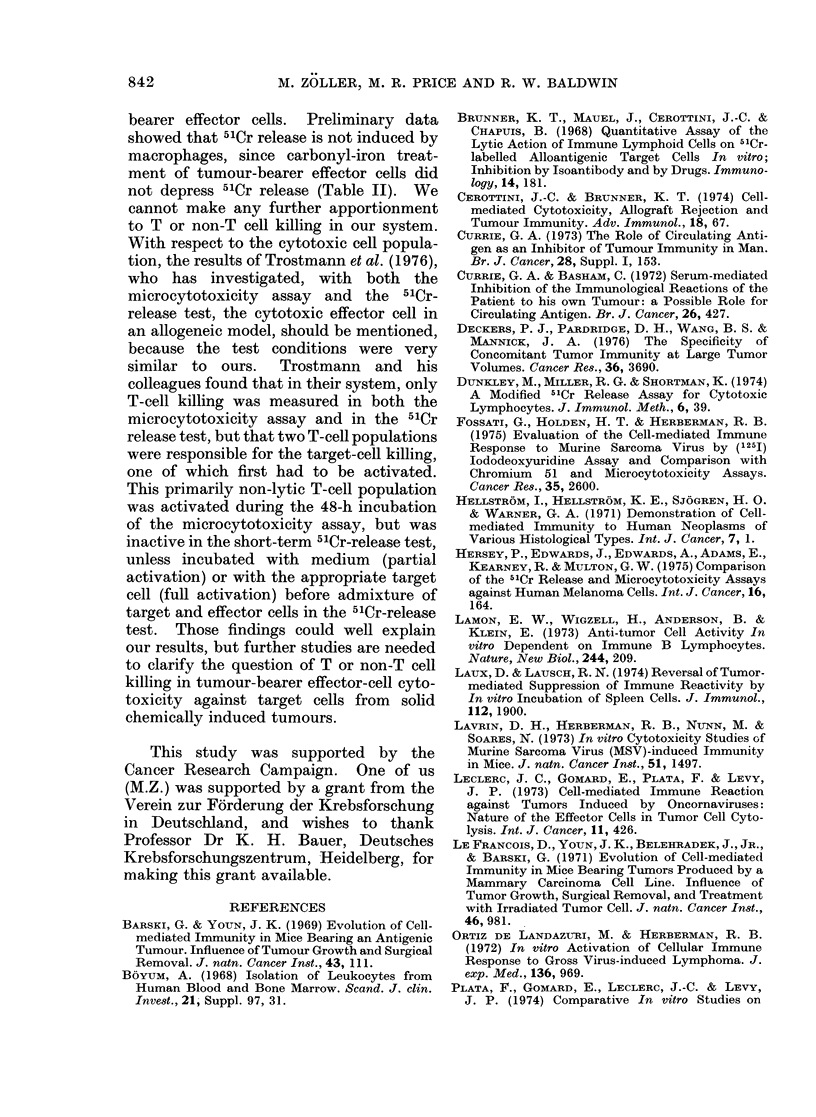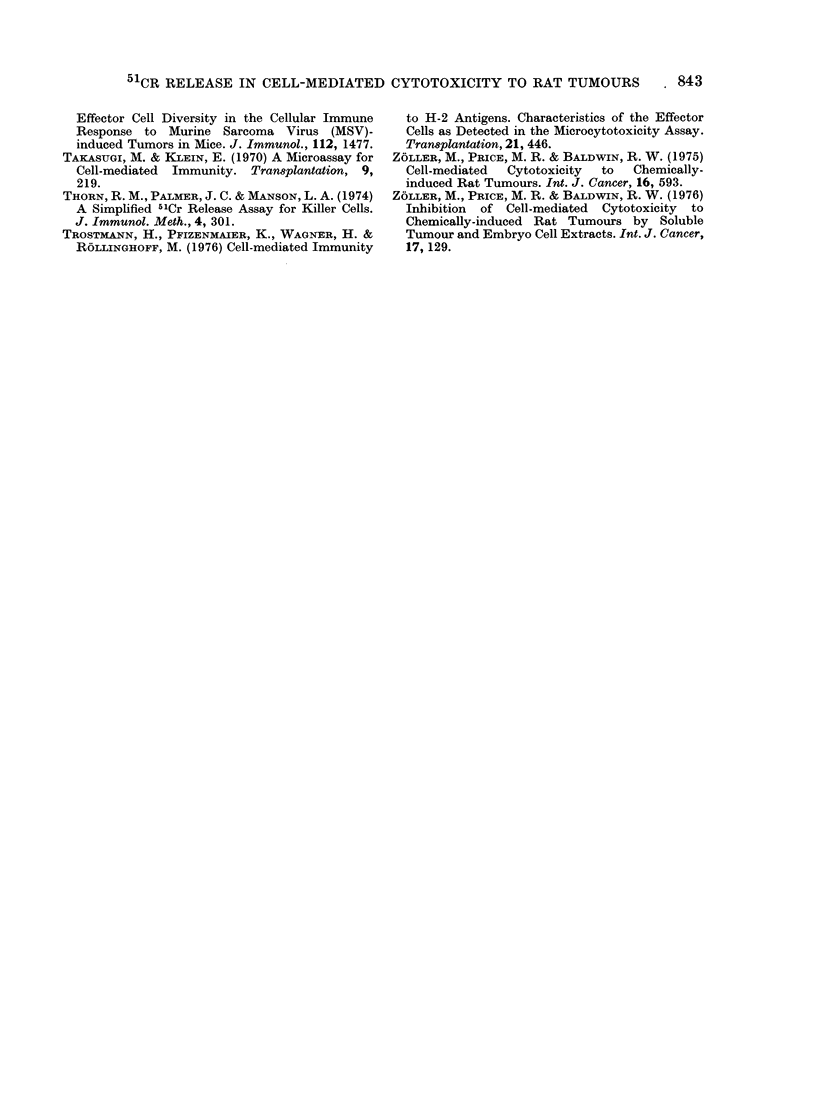# Evaluation of 51Cr release for detecting cell-mediated cytotoxic responses to solid chemically induced rat tumours.

**DOI:** 10.1038/bjc.1977.126

**Published:** 1977-06

**Authors:** M. Zöller, M. R. Price, R. W. Baldwin

## Abstract

A 51Cr-release test was developed for the detection of cell-mediated cytotoxicity against transplanted solid chemically induced rat tumours, and the findings were compared with those obtained in parallel tests using a microcytotoxicity assay. It was necessary to incorporate several modifications of the original Brunner assay (Brunner et al., 1968) in order to increase the sensitivity of the test as applied to long-term tumour lines maintained as glass-adherent cultures. These included: 1. preincubation of labelled tumour cells at 37 degrees C for 3-4 h before addition of effector cells; 2. preincubation of effector cells at 37 degrees C for 12 h before addition to target cells; 3. upon admixture of target and effector cells, intimate cell contact was established by gentle centrifugation; 4. after incubation of target and effector cells at 37 degrees C, a further incubation for 1 h at 45 degrees C was included to complete the release of 51Cr from non-viable cells. Maximal cytotoxicity of tumour-bearer effector cells was detected after 12 h incubation of lymphoid and target cells at a ratio of 200:1. Spleen cells from tumour-bearing rats during the first 2 weeks of tumour growth exhibited the same pattern of reactivity in the 51Cr-release test and the microcytotoxicity assay, although significant reduction in target-cell numbers was more frequently recorded using the microcytotoxicity assay. Tumour-bearer spleen cells showed reactivity against the homologous tumour as well as against unrelated tumours. Using either assay, pre-exposure of effector cells to 3M KC1 extracts of tumour was found to inhibit their effect on tumour cells most frequently in tests in which the effector-cell donor, soluble extract and target cell were of the same tumour line.


*Br. J. Cancer* (1977) **35**, 834.

### EVALUATION OF $^{51}$Cr RELEASE FOR DETECTING CELL-MEDIATED CYTOTOXIC RESPONSES TO SOLID CHEMICALLY INDUCED RAT TUMOURS

M. ZÖLLER,* M. R. PRICE AND R. W. BALDWIN

*From the Cancer Research Campaign Laborarories, University Park, Nottingham NG7 2RD*

Received 20 September 1976 Accepted 14 February 1977

**Summary.—A $^{51}$Cr-release test was developed for the detection of cell-mediated cytotoxicity against transplanted solid chemically induced rat tumours, and the findings were compared with those obtained in parallel tests using a microcytotoxicity assay. It was necessary to incorporate several modifications of the original Brunner assay (Brunner *et al.*, 1968) in order to increase the sensitivity of the test as applied to long-term tumour lines maintained as glass-adherent cultures. These included: 1. preincubation of labelled tumour cells at 37°C for 3–4 h before addition of effector cells; 2. preincubation of effector cells at 37°C for 12 h before addition to target cells; 3. upon admixture of target and effector cells, intimate cell contact was established by gentle centrifugation; 4. after incubation of target and effector cells at 37°C, a further incubation for 1 h at 45°C was included to complete the release of $^{51}$Cr from non-viable cells. Maximal cytotoxicity of tumour-bearer effector cells was detected after 12 h incubation of lymphoid and target cells at a ratio of 200:1.**

**Spleen cells from tumour-bearing rats during the first 2 weeks of tumour growth exhibited the same pattern of reactivity in the $^{51}$Cr-release test and the microcytotoxicity assay, although significant reduction in target-cell numbers was more frequently recorded using the microcytotoxicity assay. Tumour-bearer spleen cells showed reactivity against the homologous tumour as well as against unrelated tumours. Using either assay, pre-exposure of effector cells to 3M KCl extracts of tumour was found to inhibit their effect on tumour cells most frequently in tests in which the effector-cell donor, soluble extract and target cell were of the same tumour line.**

THE $^{51}$Cr-release test and the microcytotoxicity assay represent 2 important test systems in common use for the demonstration of lymphoid cell-mediated cytotoxic reactions against tumour cells. A major use of the $^{51}$Cr-release test in the study of tumour immunity has been with suspension cultures of tumour cells of lymphoid origin. The test is considered to generate data which reflect direct cell lysis (Cerottini and Brunner, 1974). Conversely, with the microcytotoxicity assay (Takasugi and Klein, 1970; Hellström *et al.*, 1971) cell lysis and cytostasis, as well as impairment of cell functions leading to detachment from plastic surfaces, may be measured by visual counting of remaining adherent tumour cells. Disparities between the assays may be the product of the particular assay employed (*e.g.* Plata *et al.*, 1974) or may be due to the different nature of effector cells and cytotoxic pathways operating in these tests. Evidence has been obtained that in the short-term $^{51}$Cr-release test, cytolysis is mediated by T cells (Leclerc *et al.*, 1973; Cerottini and Brunner, 1974). The situation with the microcytotoxicity assay is less well defined and both T (Fossati, Holden and Herberman, 1975) and non-T (Plata *et al.*,

* Present address: Institute of Nuclear Medicine, German Cancer Research Center, 6900 Heidelberg 1, Im Neuenheimer Feld 280, Federal Republic of Germany.

1974; Lamon *et al.*, 1973; Lavrin *et al.*, 1973) cell killing have been implicated. Furthermore, the studies of Ortiz de Landazuri and Herberman (1972) suggest that the cytotoxicity of lymphoid cells may involve *in vitro* activation of cellular immune response during incubation. Possibly, depending on the test conditions, both assays may detect T, B and non-lymphoid cell killing, which could explain that no consistent differences were detected between the 2 assays when cytotoxic responses of T- or B-enriched subfractions were tested in both assays (*e.g.* Hersey *et al.*, 1975). A further possibility is that during the 48-h incubation of target and effector cells in the microcytotoxicity assay, 2 T-cell populations may be active: cytotoxic T cells as well as initially non-lytic secondary T cells which acquire cytotoxic reactivity during the first 24 h of the test period (Trostmann *et al.*, 1976).

The present study describes an attempt to modify the Brunner $^{51}$Cr-release test (Brunner *et al.*, 1968) in order to allow evaluation of tumour-bearer lymphoid-cell-mediated cytotoxicity to cells from transplanted solid chemically induced rat tumours, cultured as long-term, glass-adherent cell lines. For comparative purposes, parallel tests were performed using the microcytotoxicity assay. The sensitivities of the 2 test systems were also compared in their ability to detect inhibition of cytotoxicity after brief exposure of effector cell preparations to extracts containing soluble tumour antigen.

### MATERIAL AND METHODS

*Target tumour cells and lymphoid cells.*—Cell cultures from the transplanted rat hepatomas D191 and D192 and sarcomas Mc7 and Mc57 were used. Details of the establishment and maintenance of these cell lines have been previously described, and the preparation of tumour-bearer lymph-node cells (LNC) and spleen cells, as well as lymphoid cells from normal donors, was also carried out according to Zöller, Price and Baldwin (1975). Lymphoid cells used in the $^{51}$Cr-release test were separated by gradient centrifugation on Ficoll–Triosil (Böyum, 1968) and were incubated for a further 12 h at 37 °C in Eagle's-HEPES buffer + 10% calf serum, washed × 5 by centrifugation with Hanks' balanced salt solution and suspended in Eagle's-HEPES + 10% calf serum. Cell viability after these procedures was greater than 90% as judged by trypan blue exclusion.

For macrophage depletion, the effector-cell population, in addition to the procedure described, was incubated with carbonyl iron for 30 min at 37 °C. After removal of ingested carbonyl iron by a magnet, a further Ficoll–Triosil gradient centrifugation was performed.

*$^{51}$Cr-labelling of target tumour cells.*—Target tumour cells ($5 \times 10^6$ to $10^7$) were labelled by incubation with 100 $\mu$Ci of [$^{51}$Cr]$Na_2CrO_4$ (Radiochemical Centre, Amersham) in a total volume of 1 to 2 ml of Eagle's-HEPES + 10% calf serum for 1 h at 37 °C. The cells were washed with Hanks' balanced salt solution and suspended in Eagle's-HEPES + 10% calf serum. After further incubation at 37 °C for 3–4 h, the cells were washed twice with Hanks' balanced salt solution and finally suspended in Eagle's-HEPES buffer + 10% calf serum.

*$^{51}$Cr-release test.*—Labelled tumour target cells (0·1 ml at $10^5$ cells/ml) were mixed with lymphoid cells (0·1 ml at $10^7$ or $2 \times 10^7$ cells/ml). Triplicate tests were performed in (9·5 × 63·5)mm stoppered polystyrene round-bottom tubes. Cells were sedimented at 300 *g* for 3 min before incubation. In preliminary tests, tubes were incubated at 37°C for 2, 4, 8, 12 and 18 h. Finally a standard incubation time of 12 h was adopted. The release of $^{51}$Cr from damaged target cells was completed by incubation at 45°C for 1 h. For the estimation of released $^{51}$Cr, phosphate-buffered saline (1·8 ml) was added to each tube and, after centrifugation at 800 *g* for 5 min, 1 ml of supernatant and 1 ml of remaining supernatant plus cell sediment were counted in a LKB-Wallac Automatic Gamma Counter. Percentage $^{51}$Cr release was calculated as follows:

$$\% \; ^{51}\text{Cr release} = \left(\frac{2 \times \text{supernatant}}{\text{supernatant} + (\text{supernatant} + \text{cell sediment})}\right) \times 100.$$

Percentage cytotoxicity was calculated using the formula:

$$\% \text{ cytotoxicity} = \frac{a - b}{c - b} \times 100,$$

where $a$ = % $^{51}Cr$ release from target cells in the presence of normal or tumour-bearer lymphoid cells, $b$ = % $^{51}Cr$ release from target cells exposed to medium alone and $c$ = % $^{51}Cr$ release from target cells lysed by incubation at 37°C in water (0·1 ml cell suspension + 1·9 ml water). The $^{51}Cr$ release from tumour cells in the presence of normal lymphoid cells compared with medium control samples was determined in each experiment. For each of the 4 tumour lines used, the mean % $^{51}Cr$ release and standard deviation was calculated as shown in Table I. Tests with tumour-bearer lymphoid cells which gave a % $^{51}Cr$ release greater than the mean % $^{51}Cr$ release plus two standard deviations for cells from that tumour line in the presence of normal lymphoid cells, were taken to represent a significant cytotoxic reaction (Table I).

TABLE I.—$^{51}Cr$ *Release from Tumour Cells in the Presence of Lymphoid Cells from Normal Rats*

| Tumour cell | Mean ± s.d. | Mean + (2 × s.d.)* % |
|---|---|---|
| Sarcoma Mc7 | 0·5±2·1 | 4·7 |
| Sarcoma Mc57 | 0·7±2·9 | 6·5 |
| Hepatoma D191 | 1·0±2·0 | 5·0 |
| Hepatoma D192 | 0·5±2·4 | 5·3 |

* These values are the lower limit for significant release of $^{51}Cr$ with tumour-bearer lymphoid cells.

*Microcytotoxicity assay.*—The microcytotoxicity assay was performed using an effector cell to target cell ratio of 1000 : 1 as previously described (Zöller *et al.*, 1975).

*Cytotoxicity inhibition test.*—3M KCl extracts of tumours were prepared according to Zöller, Price and Baldwin (1976). Lymphoid cells from normal and tumour-bearing rats were incubated at 37°C for 60 min with tumour extracts at 1 mg protein/$10^6$ cells and in control samples with Eagle's-HEPES buffer alone. The cells were sedimented by centrifugation, the supernatant was discarded, and the cells were resuspended in Eagle's-HEPES buffer prior to their use in the $^{51}Cr$-release test or microcytotoxicity assay. The percentage inhibition of cytotoxicity was calculated as follows:

$$\text{Percentage inhibition} = \frac{\begin{array}{c}\% \text{ cytotoxicity} \\ \text{of untreated} \\ \text{lymphoid cells}\end{array} - \begin{array}{c}\% \text{ cytotoxicity of} \\ \text{extract-treated} \\ \text{lymphoid cells}\end{array}}{\begin{array}{c}\% \text{ cytotoxicity of untreated} \\ \text{lymphoid cells}\end{array}} \times 100.$$

In " specific " test combinations, target cells, extract and the tumour of the effector-cell donor were derived from the same tumour line. In " cross " test combinations, the extract and the tumour of the effector-cell donor were derived from the same tumour line, but target cells were derived from a different tumour line.

### RESULTS

### *Test procedure*

Using the labelling conditions described, an uptake of [$^{51}Cr$]$Na_2CrO_4$ giving approximately 1000 ct/min/$10^3$ cells was obtained with each of the 4 tumour lines examined. In preliminary tests, when target cells were used directly after labelling, a spontaneous release of $^{51}Cr$ of up to 40–50% was observed in medium control samples after an incubation period of 12 h at 37°C. However, this was reduced to 20–35% when labelled target cells were incubated at 37°C for 3–4 h and then washed prior to a 12-h incubation at 37°C with the effector cells. In water-lysis samples, between 80 and 90% of the total $^{51}Cr$ uptake was released during 12 h.

In order to reveal cell-mediated reactivity, it was necessary to incubate lymphoid cells for 12 h at 37°C before addition to tumour target cells. The optimal ratios of effector : target cell were determined to be 200 : 1 and 100:1, since with lower numbers of lymphoid cells no cytotoxicity was detected in tumour-bearer effector-cell preparations, whereas when higher numbers of lymphoid cells were tested (at effector : target cell ratios of 500 : 1, 1000 : 1 and 2000 : 1) the release of radiolabel declined: *i.e.* the percentage release of $^{51}Cr$ from target cells in the presence of normal and tumour-bearer lymphoid cells was reduced. In such a situation it was not possible to determine meaningful values for tumour-bearer effector-cell cytotoxicity by comparison with medium control samples. As shown in Table I, at the ratios of 200 : 1 and 100 : 1, the mean $^{51}$Cr release in samples with normal lymphoid cells did not differ from medium control samples by more than approximately 1%.

Fig. 1 illustrates the results of a representative time-course study using

FIG. 1.—Development of hepatoma-D192-bearer lymphoid-cell cytotoxicity for hepatoma D192 cells with increasing incubation times of target cells with effector cells. Labelled target cells were preincubated for 4 h and effector cells were preincubated for 12 h at 37°C. The ratio of effector to target cells was 200 : 1. (△ ▲, Tumour-bearer spleen cells; ○ ●, Tumour-bearer lymph-node cells.)

hepatoma D192-bearer lymph-node and spleen cells. In this experiment, as well as in comparable time-course studies with other target-cell lines, there was little indication of significant cytotoxic reaction before at least 8 h and the effect was most pronounced after incubation for 12 h. After incubation for 18 h or longer, a high release of $^{51}$Cr was obtained in samples exposed to either tumour-bearer or normal lymphoid cells, making a calculation of cytotoxicity by comparison with the medium control samples unacceptable. When calculating the specific $^{51}$Cr release (Fig. 1) by comparison to samples with normal lymphoid cells, the loss in sensitivity was shown by a reduction of the specific $^{51}$Cr release.

No difference in the cytotoxic activities of tumour-bearer lymph-node, spleen and peritoneal cells were detected. Furthermore, the depletion of macrophages by carbonyl iron treatment did not alter the cytotoxicity significantly. This is demonstrated for D191 target and effector cells in Table II.

In all subsequent tests described, an incubation period of 12 h was employed, and spleen cells were used exclusively as effector cells at a ratio of 200 : 1 to the target tumour cells.

### *Characterization of tumour-bearer effector-cell cytotoxicity*

Using the criterion of significance defined in Material and Methods, the

TABLE II.—*Cytotoxicity of Tumour-bearer Lymph Node (LNC), Spleen (Sp), and Peritoneal Cells (PC). Effect of Macrophage Depletion (MD) (Target Cell: D192)*

| | % $^{51}$Cr release (% cytotoxicity) | | |
|---|---|---|---|
| Effector cell population* | 6 day† | 7 day† | 9 day† |
| LNC | 38·1 (18·3) | 43·4 (27·1) | 37·0 (16·6) |
| LNC: MD | 39·4 (20·5) | 43·0 (26·4) | 37·2 (16·9) |
| Sp | 37·8 (17·9) | 39·0 (19·9) | 38·2 (18·6) |
| Sp: MD | 44·5 (28·9) | 40·1 (21·7) | 39·4 (20·5) |
| PC | 42·5 (25·6) | 44·2 (28·4) | 39·1 (20·0) |
| Medium control | 26·9 | — | — |
| Lysis sample | 87·8 | — | — |

* Ratio of effector : target cell; 200 : 1.
† Effector cells harvested on the 6th, 8th and 9th days after tumour implantation.

frequency of tumour-bearer cell-mediated cytotoxic reactions was analysed in specific and cross test combinations, the results being summarized in Table III. Tumour-bearer spleen cells were taken 4–14 days after tumour implantation. From Table III it is evident that each tumour line

TABLE III.—*Frequency of Tumour-bearer Spleen-cell Cytotoxic Reactions in Specific and Cross Tests*

| Target tumour | Test combination* | No. cytotoxic reactions / No. tests (%) |
|---|---|---|
| Sarcoma | Specific | 27/34 (79) |
| Mc7 | Cross | 18/27 (67) |
| Sarcoma | Specific | 5/21 (24) |
| Mc57 | Cross | 9/17 (53) |
| Hepatoma | Specific | 17/31 (55) |
| D191 | Cross | 14/19 (74) |
| Hepatoma | Specific | 7/11 (64) |
| D192 | Cross | 8/16 (50) |

* Specific: spleen cells from donors bearing same tumour as that used as target cells. Cross: spleen cells from donors bearing a tumour different from that used as target cells.

showed broadly similar susceptibility as release of $^{51}$Cr in the presence of tumour-bearer spleen cells in either specific or cross test combinations. The highest incidence of cytotoxicity was observed using effector cells from rats bearing their tumour for 5–7 days. This is shown in specific and cross test combinations for spleen cells from D191-tumour-bearing rats (Table IV). After more than 2 weeks of tumour growth, tumour-bearer lymphoid-cell reactivity was diminished. The

TABLE IV.—*Cytotoxicity of Spleen Cells From Rats at Different Times after Implantation of Hepatoma D*191

| Days after tumour implantation | No. cytotoxic reactions / No. tests (%) Specific* | Cross* |
|---|---|---|
| 2–4 | 10/13 (77) | 3/5 (60) |
| 5–7 | 16/17 (94) | 10/13 (77) |
| 8–10 | 5/8 (63) | 6/16 (38) |
| 11–13 | 6/11 (55) | 2/8 (25) |
| 14–18 | 8/15 (53) | 2/7 (29) |

* Specific: spleen cells from rats bearing hepatoma D191 were tested against D191 target cells. Cross: D191 tumour-bearer spleen cells were tested against different target cells.

The ratio of effector : target cells was 200 : 1.

other 3 tumour lines expressed similar patterns of reactivity.

For further evaluation of tumour-bearer effector-cell cytotoxicity, spleen cells were pretreated with 3M KCl extracts of tumours, and then assayed for their cytotoxicity against tumour cells. In these experiments, effector cells were taken from rats 5–7 days after tumour implantation, and gave a basic cytotoxicity > 10%.

In specific test combinations, the mean cytotoxicity of spleen cells exposed to 3M KCl extracts was significantly less

FIG. 2.—Inhibition of tumour-bearer cell cytotoxicity for tumour cells by 3M KCl extracts of tumours. Open columns: medium-treated effector cells. Shaded columns: extract-treated effector cells. In specific tests, homologous combinations of tumour cells, effector cells and tumour extract were used. In cross tests, tumour-bearer effector cells were reacted against cells of another tumour line, and the inhibitory action of the extract from the same tumour as that borne by the lymphoid-cell donor was assessed. In parentheses: number of tests in each combination. Bars: the standard deviation of the tests.

than that of spleen cells exposed to medium alone (2·3% compared to 18·5%) (Fig. 2). Table V summarizes our experiments, showing that in 23/34 (71%) of specific tests, cytotoxicity was inhibited > than 50%. In cross test combinations, where the tumour-bearer effector cells reacted against cells of another tumour line, extracts from the same tumour as that borne by the effector-cell donor had no inhibitory effect. The mean percentage cytotoxicity of untreated and extract-treated spleen cells showed no significant difference (Fig. 2) and only in 6/35 (17%) tests was the percentage cytotoxicity inhibited > 50% (Table V).

*Comparison between the $^{51}$Cr-release test and the microcytotoxicity assay*

Table V describes the results of comparing the $^{51}$Cr-release test and the microcytotoxicity assay. With the microcytotoxicity assay, the only tests included are where the same target and effector-cell populations were used in the $^{51}$Cr-release test. For the microcytotoxicity assay an effector : target cell ratio of 1000 : 1 and for the $^{51}$Cr-release test an effector : target cell ratio of 200 : 1 was used throughout. In specific test combinations, a high frequency of cytotoxic reactions was obtained with the microcytotoxicity assay (40/43, 93%), but a lower frequency with the $^{51}$Cr-release test (56/97, 58%). In cross test combinations, again the microcytotoxicity assay was more sensitive in detecting cytotoxicity reactions (Table V).

In comparisons of the frequency of inhibition of tumour-bearer spleen-cell cytotoxicity by tumour extracts in the 2 assay systems, a positive inhibition was defined as one in which an initial cytotoxicity in medium-treated lymphoid cells was reduced > 50% by extract treatment. In specific test combinations, a high proportion of the tests were detected as inhibited in the $^{51}$Cr-release test (24/34, 71%) but inhibitory responses were more frequently detected in the microcytotoxicity assay (15/15, 100%) (Table V). In cross test combinations, the extract from the same tumour as that borne by the effector-cell donor was rarely effective in modifying tumour-bearer lymphoid-cell cytotoxicity in either assay.

### DISCUSSION

The aim of the present study was to develop a $^{51}$Cr-release test for the detection of cell-mediated immune reactions and to evaluate it for the assay of tumour-bearer spleen-cell cytotoxicity using solid chemically induced rat hepatomas and sarcomas. In preliminary tests, it was determined that the optimal incubation period for the detection of cytotoxic reactions was 12 h. With periods up to 8 h incubation, the release of $^{51}$Cr from cells exposed to tumour-bearer and to normal lymphoid cells was essentially the same. By employing incubation periods much greater than 12 h, a rapid increase of non-specifically released $^{51}$Cr from labelled tumour cells in the presence of normal and tumour-bearer effector cells

TABLE V.—*Comparison Between the $^{51}$Cr-release Test and the Microcytotoxicity Assay for the Detection of Tumour-bearer Spleen Cell Cytotoxic Reactions and Inhibition of these Reactions by 3M KCl Extracts of Tumours*

| Test combination | No. cytotoxic reactions / No. tests (%) | | No. tests inhibited by 3M KCl extract / No. tests (%) | |
|---|---|---|---|---|
| | CRT‡ | MCA‡ | CRT‡ | MCA‡ |
| Specific* | 56/97 (58%) | 40/43 (93%) | 24/34 (71%) | 15/15 (100%) |
| Cross† | 49/79 (62%) | 21/31 (68%) | 6/35 (17%) | 1/5 (20%) |

* Spleen-cell donor, extract and target cells from same tumour line.
† Spleen-cell donor and extract from same tumour line, but target cells from a different tumour line.
‡ CRT: $^{51}$Cr-release test, MCA: microcytotoxicity assay.

prevented the detection of $^{51}Cr$ release by cell-mediated cytolysis. We assume that the high unspecific release after more than 12 h incubation is probably due to nutrient deprivation, especially because in samples in which target cells were incubated with medium alone, only a slight increase in the $^{51}Cr$ release was observed.

The modifications of the assay of Brunner *et al.* (1968) adopted to maximize the sensitivity of the test, are summarized in Table VI. The preincubation of labelled target cells for 3–4 h at 37°C allowed the release of cell-surface-bound label. This was necessary in order to reduce the spontaneous release over the long incubation period of 12 h. Effector cells were preincubated for 12 h at 37°C and then washed × 5, such procedure being designed to reveal cytotoxicity in previously non-cytotoxic cell preparations, and to remove bound serum inhibitory factors (Laux and Lausch, 1974; Currie and Basham, 1972; Currie, 1973). Only small improvements were noted by this procedure, taking effector cells from tumour-bearing rats. But with effector cells from tumour-immune animals (immunized with irradiated cells and boosted with viable tumour cells) cytotoxicity was only observed after preincubation of effector cells (Zöller, unpub.). These observations are in agreement with the results of Trostmann *et al.* (1976), who found that 2 populations of T cells are involved in cell-mediated cytotoxicity, one of which first has to be activated, to exhibit a cytotoxic reaction in the usual short-term $^{51}Cr$ release. This activation can be partly achieved by preincubation of effector cells at 37°C. The second argument for preincubation of effector cells aims at the removal of bound serum inhibitory factors. This hypothesis was tested by limited papain treatment of tumour-bearer spleen cells. In fact, papain treatment resulted in a slight increase of the lytic potential of effector cells, but no further improvement could be achieved by combining both procedures (papain treatment and preincubation). We therefore assumed that, in our system, preincubation of effector cells for 12 h at 37°C is sufficient to remove possible bound serum inhibitory factors (Zöller, unpub.).

Further small improvements were achieved by intimate cell-to-cell contact (gentle centrifugation of the target-effector cell mixture, Thorn, Palmer and Manson, 1974) and by a final incubation at 45°C for 1 h, which was adopted to enhance the

TABLE VI.—*Summary of Modifications of the $^{51}Cr$-release Test*

| Modification | Effect on the release of $^{51}Cr$ from tumour cells in the presence of lymphoid cells from: Normal rats | Tumour-bearing rats |
|---|---|---|
| Target cells | | |
| Preincubation of labelled cells for 3–4 h at 37°C | Decrease | Decrease |
| Effector cells | | |
| Source (lymph node, spleen or peritoneal cavity) | None | None |
| Separation on Ficoll–Triosil | Decrease | Decrease |
| Preincubation for 12 h at 37°C | None | Increase |
| Macrophage depletion | None | Slight increase |
| Test procedure | | |
| Target : effector cell ratio | | |
| ⩽1 : 50 | None | None |
| 1 : 100 and 1 : 200 | None | Increase |
| >1 : 200 | Decrease (shielding effect) | Decrease (shielding effect) |
| Sedimentation of target and effector cells | None | Slight increase |
| Incubation time of target and effector cells : <8 h | None | None |
| 8–12 h | None | Increase |
| >12 h | Increase (nutrient depriv.) | Increase (nutrient depriv.) |
| Incubation at 45°C for 1 h after incubation at 37°C | None | Increase |

release of $^{51}$Cr from damaged target cells without inducing further tumour-cell lysis (Dunkley, Miller and Shortman, 1974).

This final version of the $^{51}$Cr-release test was compared with the microcytotoxicity assay in its ability to detect cellular cytotoxicity of tumour-bearer effector cells. Since no difference between lymph-node, spleen, and peritoneal cells was found (Table II), only the data obtained with spleen cells are presented. The model of tumour-bearer effector cells was chosen, because this system exhibited very interesting features in a previous study based on microcytotoxicity assay experiments. We found that, as early as 2 to 3 days after tumour implantation, cytotoxic values were recorded. After about 14 to 18 days, cytotoxicity was no longer demonstrable. Effector cells from tumour-bearing rats were cytotoxic in specific and cross test combinations, and it could be shown by inhibition with 3M KCl extracts of tumours and 14- to 15-day-old embryos, that tumour-bearer effector cells were sensitized against the individual specific transplantation antigen of their own tumour as well as against embryonic antigens common to all chemically induced tumours (hepatomas and sarcomas) tested (Zöller *et al.*, 1975, 1976).

The same features of reactivity as detected with the microcytotoxicity assay are now described for the $^{51}$Cr-release test. Again, a cytotoxic response was observed soon after tumour implantation. The highest incidence of cytotoxic reactions was found on Days 5 to 7 (Table III) and cytotoxicity was abolished after 14 to 18 days, depending on the tumour burden. This phenomenon has been described by other authors (Barski and Youn, 1969; LeFrancois *et al.*, 1971) and was analysed in detail by Deckers *et al.* (1976), who found that unreactivity of effector cells from animals with large tumours depends on the ratio of body weight to tumour burden. They explained the phenomenon as high-dose tolerance to tumour-associated antigens and they showed that immunological reactivity in general was unchanged. Incubation of tumour-bearer spleen cells with 3M KCl extracts of tumour, containing tumour-specific antigen, resulted in loss of cytotoxic $^{51}$Cr release in specific test combinations, but only occasionally in cross test combinations. This feature, being in full accordance with the findings obtained by the microcytotoxicity assay, may be explained by the proposal that cytolysis in specific test combinations is predominantly, but not exclusively, directed against tumour-specific antigens, thus being effectively inhibited by tumour extracts containing this antigen. The content of foetal antigens may also enable the 3M KCl extracts of tumours to inhibit cytoloysis in cross test combinations, as was occasionally observed.

The present work demonstrates that it is possible to measure cytotoxic reactions against long-term glass-adherent tumour target cells from solid chemically induced tumours with the $^{51}$Cr-release test. However, it should be noted that significant cytotoxic reactions, as well as significant inhibition of cytotoxic responses, were more frequently detected by the microcytotoxicity assay. From this we concluded that the microcytotoxicity assay is the more sensitive assay for detecting tumour-bearer effector-cell cytotoxicity against target cells from solid chemically induced tumours. But the $^{51}$Cr-release test will prove very useful where a distinction between, for example, cytolytic effects and adherence inhibition is attempted.

It is widely held that T-cell-mediated cytotoxicity accounts (at least in part) for the effects observed with both the microcytotoxicity assay and the $^{51}$Cr-release test. In the short-term $^{51}$Cr-release test, non-T-cell effects can be predominantly excluded. With respect to the microcytotoxicity assay, various effector-cell types and mechanisms are discussed. In our system of chemically induced rat hepatomas and sarcomas it is not known whether T or non-T cells are responsible for the cytotoxic reactions of tumourbearer effector cells. Preliminary data showed that $^{51}Cr$ release is not induced by macrophages, since carbonyl-iron treatment of tumour-bearer effector cells did not depress $^{51}Cr$ release (Table II). We cannot make any further apportionment to T or non-T cell killing in our system. With respect to the cytotoxic cell population, the results of Trostmann *et al.* (1976), who has investigated, with both the microcytotoxicity assay and the $^{51}Cr$-release test, the cytotoxic effector cell in an allogeneic model, should be mentioned, because the test conditions were very similar to ours. Trostmann and his colleagues found that in their system, only T-cell killing was measured in both the microcytotoxicity assay and in the $^{51}Cr$ release test, but that two T-cell populations were responsible for the target-cell killing, one of which first had to be activated. This primarily non-lytic T-cell population was activated during the 48-h incubation of the microcytotoxicity assay, but was inactive in the short-term $^{51}Cr$-release test, unless incubated with medium (partial activation) or with the appropriate target cell (full activation) before admixture of target and effector cells in the $^{51}Cr$-release test. Those findings could well explain our results, but further studies are needed to clarify the question of T or non-T cell killing in tumour-bearer effector-cell cytotoxicity against target cells from solid chemically induced tumours.

This study was supported by the Cancer Research Campaign. One of us (M.Z.) was supported by a grant from the Verein zur Förderung der Krebsforschung in Deutschland, and wishes to thank Professor Dr K. H. Bauer, Deutsches Krebsforschungszentrum, Heidelberg, for making this grant available.

### REFERENCES

BARSKI, G. & YOUN, J. K. (1969) Evolution of Cell-mediated Immunity in Mice Bearing an Antigenic Tumour. Influence of Tumour Growth and Surgical Removal. *J. natn. Cancer Inst.*, **43**, 111.

BÖYUM, A. (1968) Isolation of Leukocytes from Human Blood and Bone Marrow. *Scand. J. clin. Invest.*, **21**, Suppl. 97, 31.

BRUNNER, K. T., MAUEL, J., CEROTTINI, J.-C. & CHAPUIS, B. (1968) Quantitative Assay of the Lytic Action of Immune Lymphoid Cells on $^{51}Cr$-labelled Alloantigenic Target Cells *In vitro*; Inhibition by Isoantibody and by Drugs. *Immunology*, **14**, 181.

CEROTTINI, J.-C. & BRUNNER, K. T. (1974) Cell-mediated Cytotoxicity, Allograft Rejection and Tumour Immunity. *Adv. Immunol.*, **18**, 67.

CURRIE, G. A. (1973) The Role of Circulating Antigen as an Inhibitor of Tumour Immunity in Man. *Br. J. Cancer*, **28**, Suppl. I, 153.

CURRIE, G. A. & BASHAM, C. (1972) Serum-mediated Inhibition of the Immunological Reactions of the Patient to his own Tumour: a Possible Role for Circulating Antigen. *Br. J. Cancer*, **26**, 427.

DECKERS, P. J., PARDRIDGE, D. H., WANG, B. S. & MANNICK, J. A. (1976) The Specificity of Concomitant Tumor Immunity at Large Tumor Volumes. *Cancer Res.*, **36**, 3690.

DUNKLEY, M., MILLER, R. G. & SHORTMAN, K. (1974) A Modified $^{51}Cr$ Release Assay for Cytotoxic Lymphocytes. *J. Immunol. Meth.*, **6**, 39.

FOSSATI, G., HOLDEN, H. T. & HERBERMAN, R. B. (1975) Evaluation of the Cell-mediated Immune Response to Murine Sarcoma Virus by ($^{125}I$) Iododeoxyuridine Assay and Comparison with Chromium 51 and Microcytotoxicity Assays. *Cancer Res.*, **35**, 2600.

HELLSTRÖM, I., HELLSTRÖM, K. E., SJÖGREN, H. O. & WARNER, G. A. (1971) Demonstration of Cell-mediated Immunity to Human Neoplasms of Various Histological Types. *Int. J. Cancer*, **7**, 1.

HERSEY, P., EDWARDS, J., EDWARDS, A., ADAMS, E., KEARNEY, R. & MULTON, G. W. (1975) Comparison of the $^{51}Cr$ Release and Microcytotoxicity Assays against Human Melanoma Cells. *Int. J. Cancer*, **16**, 164.

LAMON, E. W., WIGZELL, H., ANDERSON, B. & KLEIN, E. (1973) Anti-tumor Cell Activity *In vitro* Dependent on Immune B Lymphocytes. *Nature, New Biol.*, **244**, 209.

LAUX, D. & LAUSCH, R. N. (1974) Reversal of Tumor-mediated Suppression of Immune Reactivity by *In vitro* Incubation of Spleen Cells. *J. Immunol.*, **112**, 1900.

LAVRIN, D. H., HERBERMAN, R. B., NUNN, M. & SOARES, N. (1973) *In vitro* Cytotoxicity Studies of Murine Sarcoma Virus (MSV)-induced Immunity in Mice. *J. natn. Cancer Inst.*, **51**, 1497.

LECLERC, J. C., GOMARD, E., PLATA, F. & LEVY, J. P. (1973) Cell-mediated Immune Reaction against Tumors Induced by Oncornaviruses: Nature of the Effector Cells in Tumor Cell Cytolysis. *Int. J. Cancer*, **11**, 426.

LE FRANCOIS, D., YOUN, J. K., BELEHRADEK, J., JR., & BARSKI, G. (1971) Evolution of Cell-mediated Immunity in Mice Bearing Tumors Produced by a Mammary Carcinoma Cell Line. Influence of Tumor Growth, Surgical Removal, and Treatment with Irradiated Tumor Cell. *J. natn. Cancer Inst.*, **46**, 981.

ORTIZ DE LANDAZURI, M. & HERBERMAN, R. B. (1972) *In vitro* Activation of Cellular Immune Response to Gross Virus-induced Lymphoma. *J. exp. Med.*, **136**, 969.

PLATA, F., GOMARD, E., LECLERC, J.-C. & LEVY, J. P. (1974) Comparative *In vitro* Studies on

Effector Cell Diversity in the Cellular Immune Response to Murine Sarcoma Virus (MSV)-induced Tumors in Mice. *J. Immunol.*, **112**, 1477.

TAKASUGI, M. & KLEIN, E. (1970) A Microassay for Cell-mediated Immunity. *Transplantation*, **9**, 219.

THORN, R. M., PALMER, J. C. & MANSON, L. A. (1974) A Simplified $^{51}$Cr Release Assay for Killer Cells. *J. Immunol. Meth.*, **4**, 301.

TROSTMANN, H., PFIZENMAIER, K., WAGNER, H. & RÖLLINGHOFF, M. (1976) Cell-mediated Immunity to H-2 Antigens. Characteristics of the Effector Cells as Detected in the Microcytotoxicity Assay. *Transplantation*, **21**, 446.

ZÖLLER, M., PRICE, M. R. & BALDWIN, R. W. (1975) Cell-mediated Cytotoxicity to Chemically-induced Rat Tumours. *Int. J. Cancer*, **16**, 593.

ZÖLLER, M., PRICE, M. R. & BALDWIN, R. W. (1976) Inhibition of Cell-mediated Cytotoxicity to Chemically-induced Rat Tumours by Soluble Tumour and Embryo Cell Extracts. *Int. J. Cancer*, **17**, 129.